# An essential EBV latent antigen 3C binds Bcl6 for targeted degradation and cell proliferation

**DOI:** 10.1371/journal.ppat.1006500

**Published:** 2017-07-24

**Authors:** Yonggang Pei, Shuvomoy Banerjee, Hem Chandra Jha, Zhiguo Sun, Erle S. Robertson

**Affiliations:** Department of Otorhinolaryngology-Head and Neck Surgery, and Microbiology, the Tumor Virology Program, Abramson Comprehensive Cancer Center, Perelman School of Medicine at the University of Pennsylvania, Philadelphia, Pennsylvania, United States of America; University of North Carolina at Chapel Hill, UNITED STATES

## Abstract

The latent EBV nuclear antigen 3C (EBNA3C) is required for transformation of primary human B lymphocytes. Most mature B-cell malignancies originate from malignant transformation of germinal center (GC) B-cells. The GC reaction appears to have a role in malignant transformation, in which a major player of the GC reaction is Bcl6, a key regulator of this process. We now demonstrate that EBNA3C contributes to B-cell transformation by targeted degradation of Bcl6. We show that EBNA3C can physically associate with Bcl6. Notably, EBNA3C expression leads to reduced Bcl6 protein levels in a ubiquitin-proteasome dependent manner. Further, EBNA3C inhibits the transcriptional activity of the Bcl6 promoter through interaction with the cellular protein IRF4. Bcl6 degradation induced by EBNA3C rescued the functions of the Bcl6-targeted downstream regulatory proteins Bcl2 and CCND1, which resulted in increased proliferation and G1-S transition. These data provide new insights into the function of EBNA3C in B-cell transformation during GC reaction, and raises the possibility of developing new targeted therapies against EBV-associated cancers.

## Author summary

Epstein-Barr virus (EBV) is the first characterized human tumor virus, which is associated with a broad range of human cancers. One of the latent proteins, EBV encoded nuclear antigen 3C (EBNA3C) plays a critical role in EBV-mediated B-cell transformation. Bcl6 is a master regulator required in mature B-cells during germinal center (GC) reaction. As a transcriptional repressor, Bcl6 can be targeted during malignant transformation and therefore contributes to its function as an oncoprotein during lymphomagenesis. In this study, we demonstrated that EBNA3C interacts with Bcl6 and facilitates its degradation through the ubiquitin-proteasome dependent pathway, and suppresses Bcl6 mRNA expression by inhibiting the transcriptional activity of its promoter. Furthermore, EBNA3C-mediated Bcl6 regulation significantly promotes cell proliferation and cell cycle by targeting Bcl2 and CCND1. Therefore, our findings offer new insights into the functions of EBNA3C during B-cell transformation in GC reaction and B-cell lymphoma development. This increases the possibility of developing new therapies for treating EBV-associated cancers.

## Introduction

B-cell development through the germinal center (GC) is controlled strictly by sequential activation or repression of crucial transcription factors, executing the pre- and post-GC B-cell differentiation [1]. The deregulation of induced GC reactions during B-cell development is associated with malignant transformation giving rise to different types of lymphoma and leukemia [2]. Most mature B-cell malignancies, including diffuse large B-cell lymphoma (DLBCL), follicular lymphoma (FL) and Burkitt’s lymphoma (BL) are derived from malignant transformation of GC B-cells [2,3]. Furthermore, DLBCL is the most common subtype of non-Hodgkin’s lymphoma (NHL), accounting for approximately 40% of all cases [4]. DLBCL is considered a heterogeneous group of tumors, with some specific clinicopathological variants of DLBCLs being associated with the presence of EBV [5,6].

A major regulator of the GC reaction is represented by B-cell lymphoma 6 (Bcl6), a sequence specific transcriptional repressor [7–9]. Knock-out of Bcl6 *in vivo* results in lack of GC formation and the maturation of high-affinity antibodies [10,11]. Interestingly, deregulation of Bcl6 expression can be found in BL, FL and DLBCL [12,13]. In addition, Bcl6 is the most frequent oncogene involved in roughly 40% of the cases of DLBCLs, and its locus is frequently rearranged due to chromosomal translocations in DLBCL [14,15]. As a key transcriptional repressor in normal B-cell differentiation, Bcl6 was shown to repress NF-κB and the positive regulatory domain I element (PRDM1) also known as Blimp-1 in DLBCLs [16–18]. Also, Bcl6 is now been investigated as a potential therapeutic target for the treatment of tumors with rationally designed specific Bcl6 inhibitors [19–21].

EBV is a lymphotropic virus that is linked to many kinds of B-cell malignancies, including BL, FL and DLBCL [22,23]. EBV infection transforms primary human B-cells into continuously growing lymphoblastoid cells (LCLs) and different latent types were established in EBV-infected cells [23,24]. During latency III or the growth program, EBV expresses the full complement of oncogenic latent proteins, including EBV nuclear antigens EBNA1, EBNA2, EBNA3A, EBNA3B, EBNA3C and EBNA-LP, as well as latent membrane proteins LMP1, LMP2A and LMP2B in addition to numerous RNAs and miRNAs [25]. Genetic studies using recombinant virus strategies demonstrated that EBNA1, EBNA2, EBNA3A, EBNA3C, EBNA-LP and LMP1 are essential or very important for EBV-mediated transformation of primary B-cells *in vitro* [26–28]. Specifically, EBNA3C has the ability to function as a transcriptional activator and repressor, and can regulate the transcription of both cellular and viral genes [29,30]. A number of earlier studies have shown that EBNA3C interacts with a wide range of transcription factors and modulators, such as c-Myc [31], IRF4 [32], CtBP [33], p53 [34], E2F1 [35] and E2F6 [36], which leads to dysregulation of their associated cellular functions.

Previous studies have indicated that expression of EBV latent proteins were associated with Bcl6 expression [37–39]. For example, in some B-cell lymphomas, Bcl6 expression was inversely correlated with LMP1 expression, and some data suggested that LMP1 can cause downregulation of Bcl6 [6,37]. However, the link between LMP1 and Bcl6 was not fully explained as Bcl6 expression was also inhibited in LMP1-negative cells [38]. Similar studies have shown that LMP1 through heterologous expression was unable to suppress expression of Bcl6 in DLBCLs [39]. In addition, EBNA2 can interfere with the germinal center phenotype by downregulating Bcl6 in Non-Hodgkin's Lymphoma cells [39]. Furthermore, EBV encoded microRNAs can repress Bcl6 expression in DLBCL [38]. However, the mechanism by which Bcl6 is down-regulated in EBV-infected cells is still not fully understood. Our goal is to determine the role of EBNA3C in regulating expression of Bcl6 oncoprotein in B-cells, and further uncover novel molecular mechanisms by which EBNA3C-mediated regulation of cellular functions can lead to B-cell transformation.

## Results

### EBNA3C down-regulates Bcl6 expression in EBV-infected peripheral blood mononuclear cells

**Fig 1:**
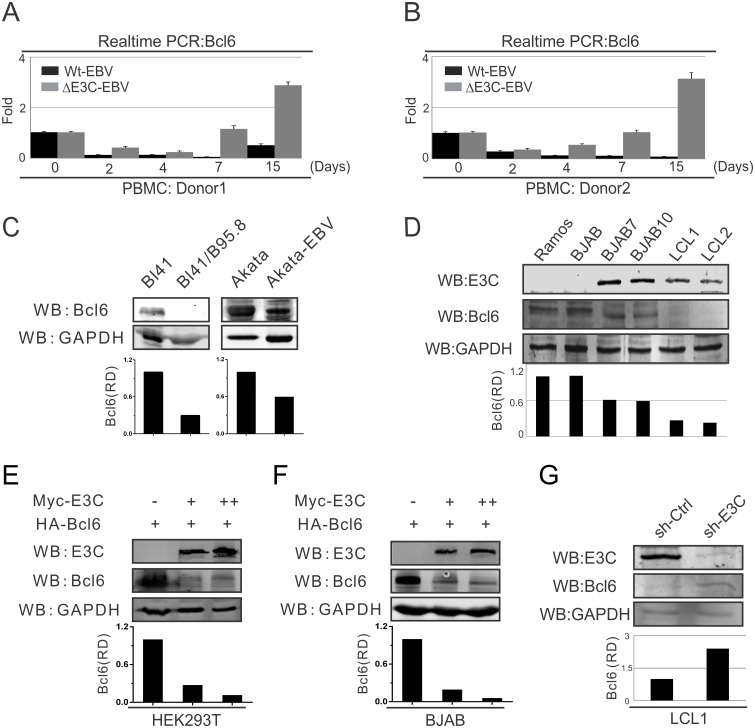
EBNA3C down-regulates Bcl6 expression in EBV-infected PBMCs. A-B) 10 million human peripheral blood mononuclear cells (PBMC) from donor 1 and donor 2 were infected with BAC-GFP wild-type EBV or ΔE3C-EBV for 4 hours. Cells were harvested at indicated time points, then total RNA was isolated and subjected to cDNA preparation according to the manufacture's instruction followed by quantitative Real-time PCR for detecting Bcl6 transcription levels. C) 10 million Burkitt’s lymphoma (BL) cells BL41 or Akata and EBV-positive BL41/B95.8 or Akata-EBV cells were lysed with RIPA buffer and western blot analysis was performed with indicated antibodies. The relative density (RD) of Bcl6 protein was quantified and shown. D) 10 million EBV-negative Ramos, BJAB; EBNA3C stably expressed BJAB7, BJAB10; EBV-transformed LCL1, LCL2 cells were harvested and total cell lysates were subjected to western blot analysis using indicated antibodies. E-F) 10 million E) HEK293T and F) Saos-2 cells were transfected in a dose-dependent manner with increasing amounts EBNA3C constructs and western blot analysis was performed using specific antibodies as indicated. G) Lentivirus mediated stable EBNA3C knock-down (sh-E3C) or scramble control (sh-Ctrl) LCL1 cells were subjected to western blot analysis with indicated antibodies. Protein bands from western blot were analyzed by the Odyssey imager software and represented as bar diagrams based on internal loading control GAPDH. These results shown are representative of three independent experiments.

To determine the expression levels of Bcl6 during EBV infection of primary B-cells, 10 million human peripheral blood mononuclear cells (PBMCs) from two donors, respectively, were infected with wild-type BAC-GFP-EBV or EBNA3C-deleted (ΔE3C) BAC-GFP-EBV. The infected cells were harvested at different time points (0, 2, 4, 7 and 15 days post-infection), then mRNA was extracted and Real-time PCR was performed for Bcl6 detection. The results showed that for both donors Bcl6 expression was down-regulated and expressed at a relatively low level after wild-type EBV infection. However, its expression was consistently enhanced with the EBNA3C-deleted EBV infection as much as 20–35 fold over wild-type infection (Fig 1A and 1B). This suggested that EBNA3C can play a key role in Bcl6 expression during EBV infection.

To determine the effect of EBNA3C on Bcl6 expression in Burkitt’s lymphoma cells, western blot analysis was also performed in EBV-negative BL41 and Akata cells, as well as the corresponding EBV-positive BL41/B95.8, Akata-EBV cells. We found a significant downregulation of Bcl6 expression in the presence of EBV-infected BL41 and Akata cell lines of approximately 2–4 fold (Fig 1C). To further investigate whether the differential expression was due to the presence of EBNA3C, EBV-negative Ramos and BJAB cells; EBNA3C expressing stable BJAB cells (BJAB7 and BJAB10); and EBV-transformed lymphoblastoid cell lines (LCL1 and LCL2), were analyzed by western blot. Similarly, Bcl6 protein expression was down-regulated close to 50% in the presence of EBNA3C in Burkitt’s lymphoma cells and were dramatically suppressed in EBV-transformed LCLs (Fig 1D). These results strongly suggested that EBNA3C contributes to inhibition of Bcl6 expression.

To further examine if Bcl6 expression was regulated by EBNA3C specifically, HEK293T and BJAB cells were transfected with a dose-dependent increase of EBNA3C in addition to heterologous expression of Bcl6. The western blot results demonstrated that EBNA3C expression led to a strong reduction in Bcl6 protein expression in human cells, including B-cell lines of about 4–10 fold (Fig 1E and 1F). To further explore the role of EBNA3C in modulating Bcl6 expression levels in EBV transformed LCLs, EBNA3C knocked-down LCL1 cell line was generated with specific EBNA3C short hairpin RNA (sh-E3C) [40]. Compared to the control LCL1 (sh-Ctrl), Bcl6 protein expression was significantly increased by at least 2-fold in sh-E3C LCL1 cells (Fig 1G). The results provide additional supporting evidence demonstrating that down-regulation of Bcl6 expression can be specifically linked to EBNA3C.

### EBNA3C physically associates in a molecular complex with Bcl6 in human cells

**Fig 2:**
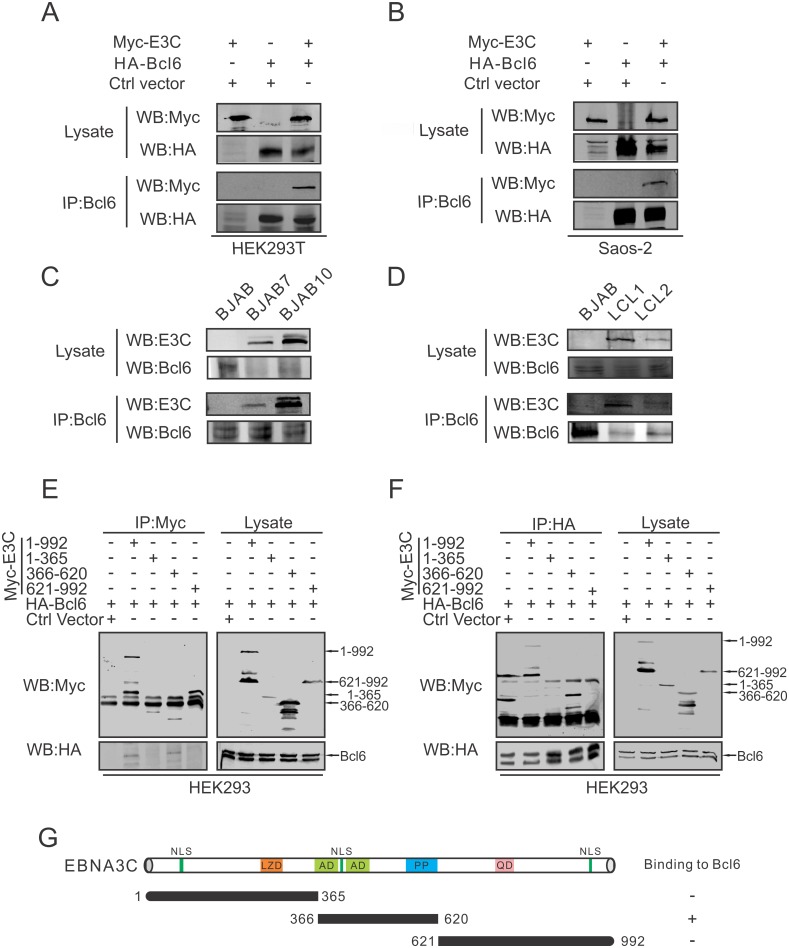
EBNA3C associates with Bcl6 in human cells. A-B) 10 million A) HEK293T and B) Saos-2 cells were transfected with Myc-tagged EBNA3C or HA-tagged Bcl6. Empty vectors were used for balance the total DNA per transfection. Transfected cells were harvested 48 hours post-transfection and approximately 5%-10% of the lysates were used as input and the residual lysates were immunoprecipitated (IP) with 1μg anti-Bcl6 antibody. Lysate and immunoprecipitated samples were resolved by 10% SDS-PAGE and western blot analysis was performed using the indicated antibodies. C-D) 30 million BJAB, BJAB7, BJAB10, LCL1 and LCL2 cells were lysed and co-immunoprecipitation was performed with Bcl6 specific antibody. Immunoprecipitated samples were resolved by 10% SDS-PAGE and endogenous EBNA3C and Bcl6 were detected by their specific antibodies. E-F) 10 million HEK293 cells were transfected with either control vector or full length and different truncated mutants of Myc-tagged EBNA3C combined with HA-tagged Bcl6 construct. At 48 hours post-transfection, cells were harvested and immunoprecipitation was performed with 1μg E) anti-Myc or F) anti-HA antibody. IP samples were resolved in 10% SDS-PAGE, and western blots were performed using anti-Myc and anti-HA antibodies. G) The schematic diagram summarizes the binding domains between different regions of EBNA3C and Bcl6. NLS, nuclear localization signal (aa 72–80, 412–418 and 939–945); LZD, leucine zipper domain; AD, acidic domains; PP, Proline-rich; QP, glutamine-proline-rich. +, binding; -, no binding.

Next, we examined whether EBNA3C could interact directly with Bcl6. Two experiments using Co-Immunoprecipitation (Co-IP) assays were performed in different cell types. First, HEK293T cells were transfected with Myc-tagged EBNA3C and HA-tagged Bcl6. The Co-IP results showed that EBNA3C associated in a complex with Bcl6 (Fig 2A). Similarly, an experiment using Saos-2 cells also showed the formation of a complex of EBNA3C and Bcl6 in these cells (Fig 2B). Second, to further determine the interaction in B-cell lines, BJAB, EBNA3C stably expressed BJAB cells (BJAB7 and BJAB10), and EBV-transformed cells (LCL1 and LCL2) were used in our Co-IP experiment. Western blot analysis also validated the above results demonstrating that endogenous EBNA3C can physically associate with Bcl6 in the background of B-cells and more importantly in EBV-transformed lymphoblastoid cell lines (Fig 2C and 2D).

To determine the functional binding domain of EBNA3C that specifically interacts with Bcl6, Myc-tagged full length and truncated regions of EBNA3C (1-365aa, 366-620aa, 621-992aa) were co-transfected into HEK293T cells with HA-tagged Bcl6. The targeted protein was immunoprecipitated with anti-Myc or anti-HA antibody, respectively. The results demonstrated that Bcl6 was associated with EBNA3C (366-620aa) along with the full-length EBNA3C protein (1-992aa) (Fig 2E and 2F). Little or no detectable co-immunoprecipitation was observed with the control vector indicating the specificity of the complex between EBNA3C and Bcl6. These results showed that EBNA3C amino acid residues 366-620aa which includes the acidic domain were responsible for the interaction of EBNA3C and Bcl6 protein (Fig 2G).

### EBNA3C co-localizes to nuclear compartments with Bcl6 in human cells

**Fig 3:**
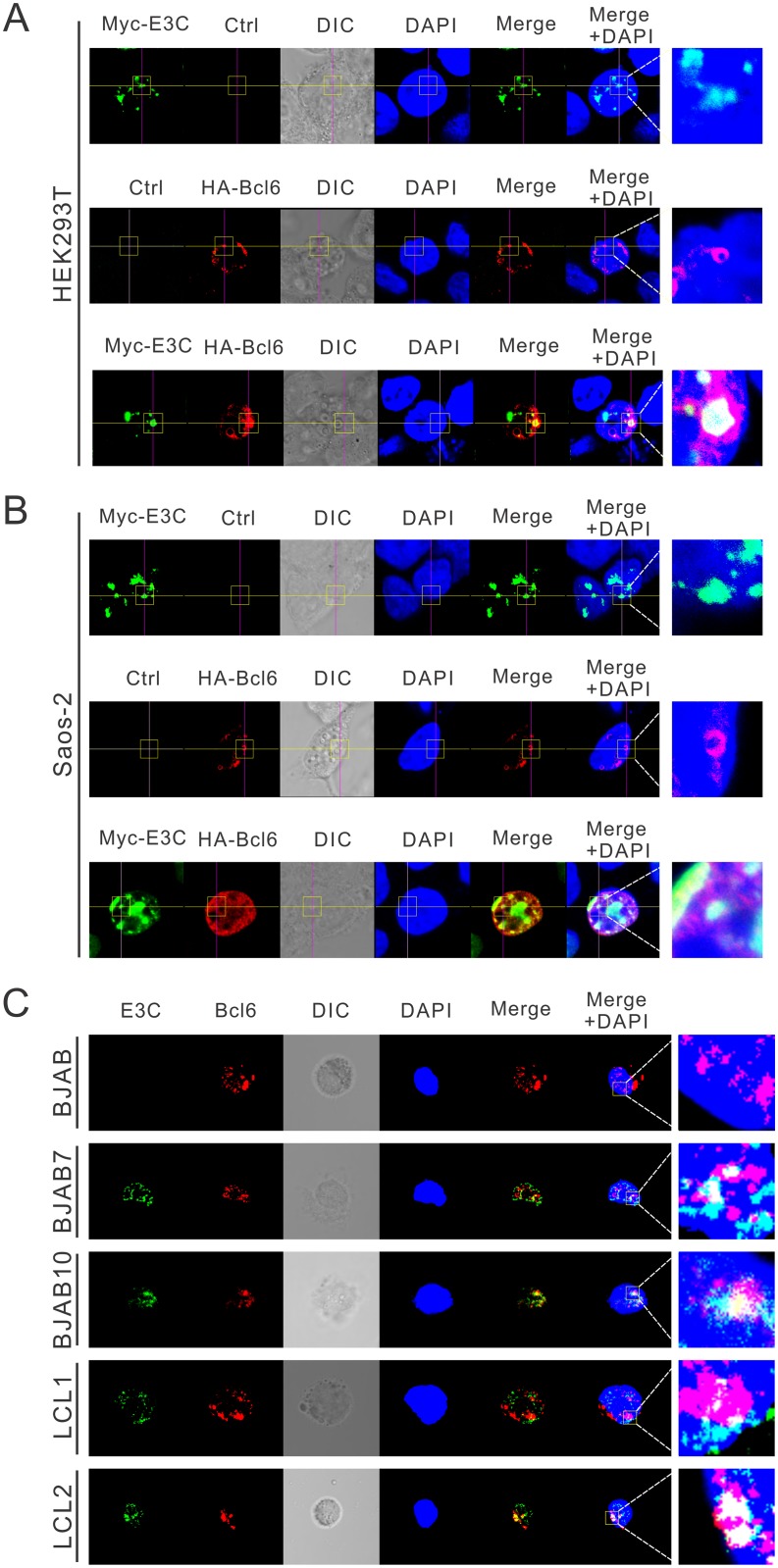
Co-localization of EBNA3C and Bcl6 in human cells. A-B) 0.1 million A) HEK293T or B) Saos-2 cells were plated on coverslips and transfected with Myc-EBNA3C or HA-Bcl6 using jetPRIME transfection reagent. C) BJAB, BJAB7, BJAB10, LCL1 and LCL2 cells were plated on slides and air-dried. All the cells were fixed with 4% PFA, stained with antibodies. Ectopic and endogenous expression of EBNA3C were detected by mouse anit-Myc (9E10) or mouse anti-EBNA3C (A10) antibodies, respectively, followed by the secondary anti-mouse Alexa Fluor 488 (green). Ectopic and endogenous expression of Bcl6 were detected by rabbit anti-Bcl6 antibodies, followed by anti-rabbit Alexa Fluor 594 (red) as the secondary antibody. The nuclei was subsequently stained with DAPI, and the images were captured using an Olympus Fluoview confocal microscope. These results shown are representative of three independent experiments.

Previous studies have shown that EBNA3C binds to Bcl6 specifically in human cells, so it is expected that these two proteins would be localized within the same cellular compartments. To determine the co-localization of EBNA3C and Bcl6, HEK293T cells were transfected with constructs expressing Myc-tagged EBNA3C and HA-tagged Bcl6, and the cellular localization of the expressed proteins was studied using immunofluorescence microscopy. In cells transfected independently with Myc-EBNA3C or HA-Bcl6 alone, both were found to be primarily expressed in the nucleus (Fig 3A). In cells co-transfected with Myc-EBNA3C and HA-Bcl6, the merged yellow fluorescence demonstrated that EBNA3C co-localized with Bcl6 in human cells (Fig 3A). Similar results were also observed in Saos-2 cells (Fig 3B).

To further determine the co-localization of EBNA3C and Bcl6 proteins in more relevant B-cells, immunofluorescence assays were performed using antibodies specific to EBNA3C and Bcl6 proteins in order to examine the endogenous expression in different B-cell lines. The results further confirmed that EBNA3C co-localized with Bcl6 in nuclear compartments of EBV-transformed LCLs (Fig 3C). This was consistent with the results of the above studies which demonstrated that EBNA3C associated with Bcl6 in nuclear complexes as seen in the Co-IP experiments in human cells.

### Bcl6 expression is regulated by EBNA3C via the ubiquitin-proteasome dependent pathway

**Fig 4:**
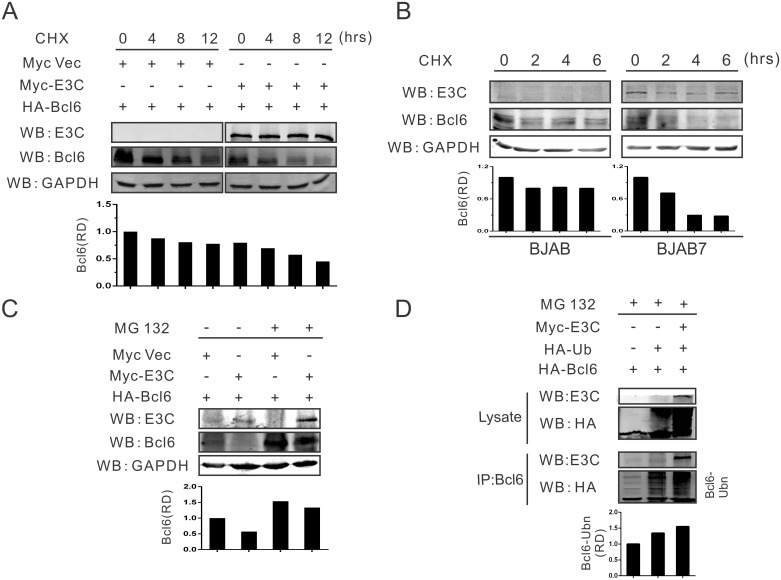
Bcl6 expression is regulated by EBNA3C via the ubiquitin-proteasome dependent pathway. A) 10 million HEK293T cells were transfected with HA-Bcl6 in combination with Myc-EBNA3C or control vector, treated with protein synthesis inhibitor cycloheximide (CHX) after 24 hours transfection as 40 μg/ml concentration. Cells were incubated with CHX for indicated times (0, 4, 8, 12 hours), and lysed with RIPA buffer, then cell lysates were quantitated and used for western blot analysis with indicated antibodies. B) 10 million BJAB or BJAB7 cells were incubated with CHX for 0, 2, 4, 6 hours, harvested and lysed with lysis buffer. Protein samples were resolved by 10% SDS-PAGE. Western blot analysis was performed by specific antibodies as shown. C) 10 million HEK293T cells were transfected with HA-Bcl6 and Myc-EBNA3C or control vector. After 24 hours transfection, cells were treated with MG132 for 16 hours and harvested, then western blot analysis was performed with indicated antibodies. D) 10 million HEK293T cells were transfected with the indicated constructs, incubated with MG132 for another 12 hours after 24 hours transfection, collected and detected with western blot using specific antibodies. The relative intensity (RD) of ubiquitinated Bcl6 complex was quantified and shown.

To explore the potential mechanism of EBNA3C-mediated down-regulation of Bcl6, a stability assay was performed to determine whether EBNA3C regulated Bcl6 expression at the protein level. HEK293T cells were transfected with HA-tagged Bcl6 and Myc-tagged EBNA3C or Myc-tagged empty vector. Twenty-four hours post-transfection, cells were incubated with protein synthesis inhibitor cycloheximide (CHX) and monitored for Bcl6 protein levels at 0, 4, 8, 12 hours by western blot analysis. As expected, the results showed that the stability of Bcl6 protein was significantly decreased by greater than 50% in the presence of EBNA3C by 12 hours, while the Bcl6 protein level was more stable in the absence of EBNA3C (compare right and left panels, Fig 4A). To further support these results, BJAB (EBNA3C negative B-cells), and BJAB7 (BJAB stably expressing EBNA3C cells) were treated with CHX for 0, 2, 4, and 6 hours. The following western blot analysis also demonstrated that there was a dramatic reduction in the stability of Bcl6 protein which was directly associated with EBNA3C expression as seen by the significant change in the Bcl6 levels by 2 hours post cycloheximide treatment and greater than 50% by 6 hours (compare right and left panels, Fig 4B).

Bcl6 expression is strictly regulated during GC reaction, and its degradation through the ubiquitin-proteasome pathway is crucial for B-cell development or lymphomagenesis in temporal function. Earlier studies showed that Bcl6 could be degraded by the ubiquitin-mediated proteasome [41,42]. Therefore, it is expected that Bcl6 degradation is likely mediated by EBNA3C utilizing a similar pathway as EBNA3C has been shown to recruit E3 ligases for targeted degradation of cellular substrates [43,44]. To determine whether this is the case, HA-Bcl6 was transfected along with Myc-EBNA3C or control vector. Twenty four hours post-transfection, the cells were treated with the proteasome inhibitor MG132 for 12 hours or vehicle control. The following western blot analysis showed that EBNA3C promoted the degradation of Bcl6 protein, which was similar to the above results. However, Bcl6 protein expression was increased after MG132 incubation, even in the presence of EBNA3C (Fig 4C). These results demonstrated that the stability of the Bcl6 protein is regulated by EBNA3C via the ubiquitin-proteasome pathway.

To further support our hypothesis, ubiquitination assays were performed with different expression plasmids for Myc-E3C, HA-Ub and HA-Bcl6, and incubated for 24 hours followed by MG132 treatment for another 12 hours. This was followed by immunoprecipitation and western blot analysis. The results demonstrated enhanced ubiquitination of Bcl6 when EBNA3C was expressed, when compared with control vector or HA-Ub alone (Fig 4D). This strongly indicated that Bcl6 is likely degraded by expression of EBNA3C through the ubiquitin-proteasome dependent pathway.

### EBNA3C regulates Bcl6 mRNA expression through inhibition of its promoter activity

**Fig 5:**
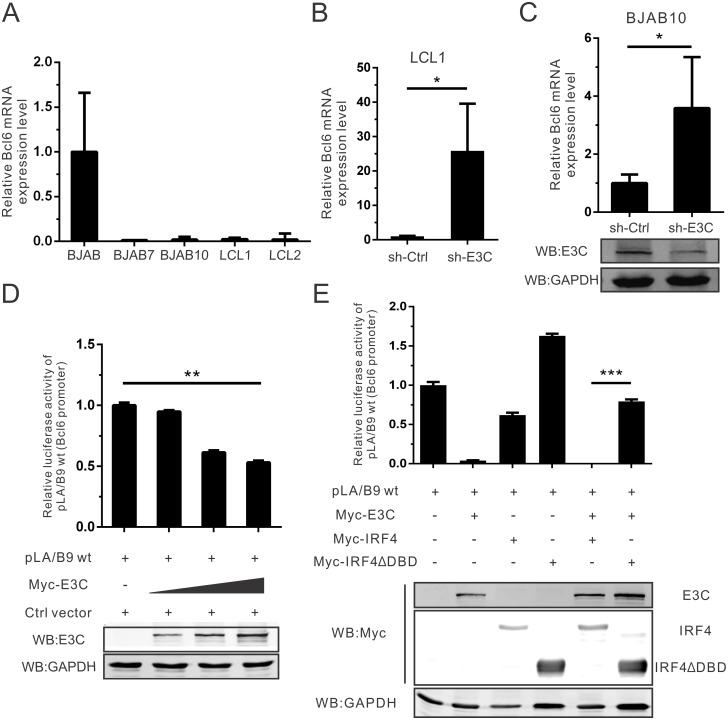
EBNA3C regulates Bcl6 mRNA expression through inhibition of its promoter activity. A) 5 million BJAB, BJAB7, BJAB10, LCL1 and LCL2 cells were harvested and extracted total RNA using Trizol reagent. Then cDNA was prepared with reverse transcriptase kit, and detected Bcl6 mRNA expression by quantitative Real-time PCR analysis (SYBR green). GAPDH was set as an internal reference. Each sample was determined in triplicate. B) EBNA3C knock-down (sh-E3C) stable LCL1 or control (sh-Ctrl) LCL1 cells were harvested and Bcl6 mRNA expression was detected using Real-time PCR as mentioned. C) 10 million BJAB10 cells were transfected with specific EBNA3C (sh-E3C) or control (sh-Ctrl) short hairpin RNA. At 48 hours post-transfection, total RNA was extracted, reverse-transcribed, followed by quantitative Real-time PCR analysis. Meanwhile, EBNA3C expression was also detected by western blot analysis. D) HEK293T cells were transfected with the reporter constructs containing wild-type Bcl6 promoter (pLA/B9) and increasing amount of Myc-EBNA3C. Cells were collected and lysed in lysis buffer at 48 hours post-transfection. Luciferase activity was measured according to the dual-luciferase reporter assay kit. Mean values and standard deviations of two independent experiments were presented. Cell lysate was resolved by 10% SDS-PAGE in order to check EBNA3C expression. GAPDH western blot was done as an internal loading control. E) HEK293T cells were transfected with wild-type Bcl6 promoter reporter plasmids in combination with different expression constructs as indicated. Cells were collected and lysed, then the lysate were used to detect luciferase activity as previously described.

Bcl6 gene expression is tightly regulated during mature B-cell development [2,45,46]. Our above studies showed that Bcl6 mRNA expression was down-regulated after EBV infection, and that this was associated with EBNA3C expression. To further define how EBNA3C can regulate Bcl6 expression at the mRNA level, different B-cell lines (BJAB, BJAB7, BJAB10, LCL1 and LCL2) were used to monitor endogenous Bcl6 mRNA expression. Bcl6 mRNA expression was significantly greater (>20 fold) in BJAB cells compared to EBNA3C stably expressed BJAB7 and BJAB10 cell, and also EBV-transformed LCL1 and LCL2 cells (Fig 5A). To verify that the inhibition was related to the presence of EBNA3C, EBNA3C stably knocked-down LCL1 (sh-E3C) and the control LCL1 (sh-Ctrl) were used to detect Bcl6 mRNA expression. The results showed that Bcl6 mRNA expression was upregulated significantly after the knockdown of EBNA3C (Fig 5B). In addition, BJAB10 cells were then transfected with specific EBNA3C short hairpin RNA (sh-E3C) to knock down EBNA3C expression. Expectedly, Bcl6 mRNA expression was increased in the EBNA3C knockdown cell lines (Fig 5C). These findings undoubtedly provide new evidence that EBNA3C can inhibit Bcl6 mRNA expression.

Bcl6 promoter transcriptional activity could not only be controlled by Bcl6 through binding to the upstream regulatory region of its gene [15], but is also inhibited directly by the transcription factor IRF4 via binding to multiple sites within its promoter [47]. To test whether EBNA3C-mediated Bcl6 mRNA down-regulation was related to its transcriptional activity at the Bcl6 promoter, a dual-luciferase reporter system was implemented. The reporter construct containing a wild-type Bcl6 promoter (pLA/B9) and a dose-dependent increase of Myc-EBNA3C were transfected into cells. Meanwhile, the thymidine kinase promoter-Renilla luciferase reporter plasmid (pRL-TK) was additionally transfected and used as a control for transfection efficiency. The luciferase assay results clearly revealed that the Bcl6 promoter activity was inhibited by EBNA3C in a dose-dependent manner (Fig 5D).

Previous experiments showed that EBNA3C did not bind with DNA directly and functions through binding of other cellular transcription proteins to regulate gene expression [48]. Therefore, other transcription proteins mediate the inhibition of viral and cellular genes. EBNA3C interacted with p53, attenuated its function and mediated its degradation [34,44,49]. Furthermore, p53 can activate Bcl6 transcription [50]. It suggests that the transcription activity of the Bcl6 promoter may be inhibited by EBNA3C-induced p53 degradation. However, our results using MEF(p53-/-) cells showed that the regulation of Bcl6 promoter by EBNA3C was independent of the function of p53 protein (S1 Fig). Among several other transcriptional proteins that inhibited Bcl6 promoter, we found that IRF4, a DNA-binding protein, was an important transcription factor for regulating Bcl6 promoter activity [47]. Interestingly, EBNA3C also interacted with IRF4 and contributed to stabilization of IRF4 [32]. One study showed that a high level of IRF4 was expressed in LMP1-KO EBV-induced lymphoma [51]. Thus, we further assessed the possible function of EBNA3C on IRF4-mediated Bcl6 promoter activity. To specifically test the Bcl6 promoter activity, the wild-type and DNA binding domain (DBD)-deleted IRF4 plasmids were used (Fig 5E). The results showed that EBNA3C enhanced the IRF4-mediated inhibition of the Bcl6 promoter activity, and the effect was dependent on the DNA binding domain of IRF4 as the promoter repression was rescued when EBNA3C and IRF4-ΔDBD were co-expressed (Fig 5E). It also suggested that IRF4 is one of the major transcription factors that mediate EBNA3C-regulated inhibition of Bcl6 promoter activity.

### EBNA3C promotes cell proliferation by upregulating Bcl6-targeted Bcl2 expression

**Fig 6:**
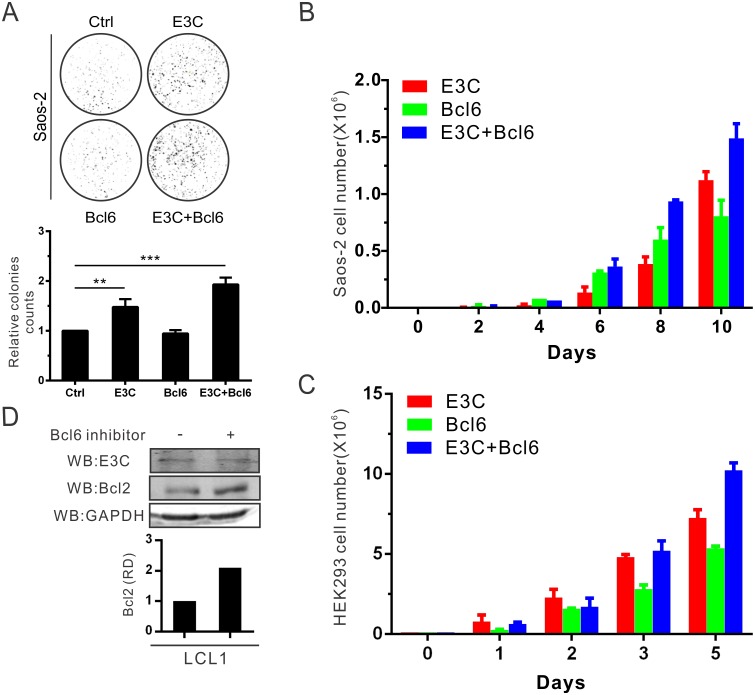
EBNA3C promotes cellular proliferation by upregulating Bcl6-targeted Bcl2 expression. A) Saos-2 cells were transfected with the indicated plasmids in combination with the GFP vector and selected with G418 antibiotic for 2 weeks. The GFP fluorescence of each plate was scanned by PhosphorImager and the colonies were calculated by Image J software. B) 10^5^ selected cells from the former experiment were plated and cultured for 10 days. Viable cells were counted at the indicated time using trypan blue staining. C) HEK293 cells were transfected, selected and counted the numbers. D) 10 million LCL1 cells were treated with 50 μg/ml (110 μM) Bcl6 inhibitor for 15 hours. Then cells were harvested, and western blot analysis was performed with indicated antibodies. These results shown are representative of three independent experiments.

To examine the effect of EBNA3C on Bcl6-mediated cell proliferation, Saos-2 cells were transfected with expression constructs of EBNA3C and Bcl6, and selected with G418 for two weeks to monitor colony formation. We observed a significant increase in colony numbers when EBNA3C and Bcl6 were co-transfected in comparison to those transfected with only EBNA3C or Bcl6 (Fig 6A). We further extended these studies by performing cell proliferation assays as determined by cell counting for 10 days in Saos-2 cells (Fig 6B). A similar experiment was also repeated in HEK293 cells (Fig 6C). These results demonstrated that expression of EBNA3C and Bcl6 results in a strong induction in cell proliferation.

The anti-apoptotic proto-oncogene Bcl2 protein is a critical regulator protein and its expression is inhibited by Bcl6 in GC B-cells [52,53]. Therefore, it was reasonable to believe that EBNA3C-mediated Bcl6 down-regulation will lead to up-regulation of Bcl2 expression. Therefore, its anti-apoptotic function will be activated and leads to promotion of cell proliferation. To determine the expression of Bcl2 in B-cells, LCL1 was treated with a Bcl6-specific inhibitor (79–6) to suppress Bcl6 activity [20]. The Bcl6 inhibitor disrupts Bcl6 transcription activity by binding to its BTB/POZ domain [20]. The following western blot results showed that Bcl2 expression was up-regulated after Bcl6 inhibitor incubation in B-cells by approximately 2-fold (Fig 6D). To further support the results, Bcl2 mRNA expression was determined in the stable EBNA3C or Bcl6 knock-down LCL1 cells to verify that EBNA3C promoted Bcl2 up-regulation through Bcl6 down-regulation in LCLs (S2 Fig). This suggests that EBNA3C-mediated Bcl6 inhibition can contribute to cell proliferation through the Bcl2-associated signaling pathway.

### Bcl6 knockdown leads to increased transforming activity in LCLs

**Fig 7:**
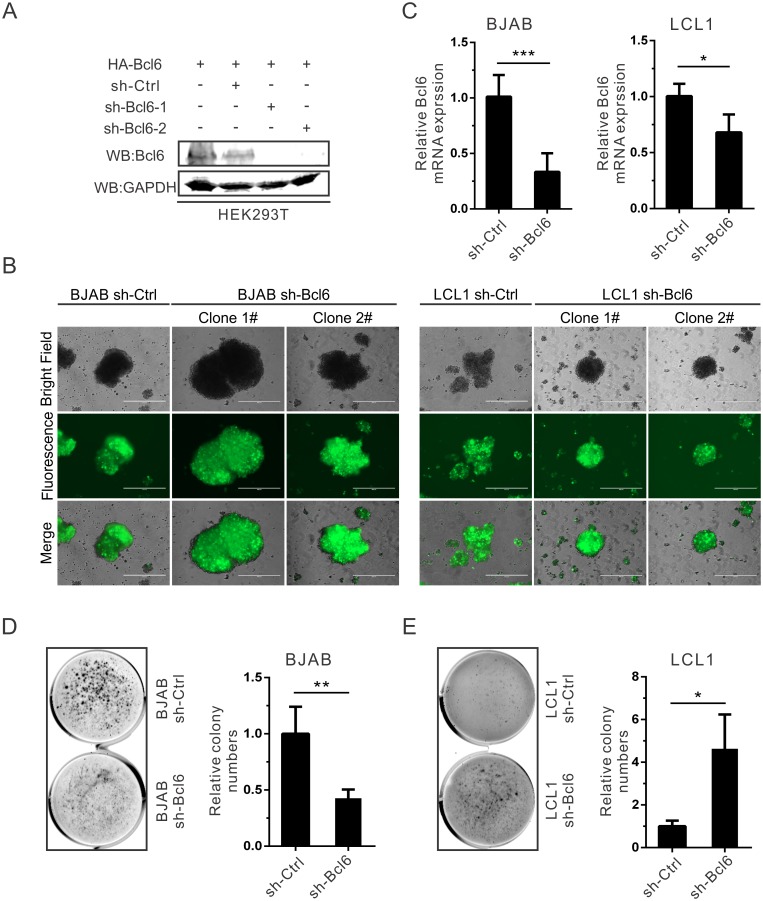
Bcl6 knockdown leads to increased transforming activity in LCLs. A) HEK293T cells were transfected with sh-Ctrl, sh-Bcl6-1 or sh-Bcl6-2 plasmid in combination with HA-tagged Bcl6. After 48 hours transfection, cell were collected, lysed and detected with western blot. B-C) BJAB or LCL1 cells were infected using lentiviruses and selected by puromycin for at least two weeks. The selected cells were monitored the GFP fluorescence (B) and Bcl6 mRNA expression with Real-time PCR (C), respectively. D-E) Bcl6 knock-down BJAB (D) or LCL1 (E) cells was assessed for their ability to promote colony formation using the soft agar assays. Photomicrograph of representative colonies from three independent experiments are shown.

The soft agar assay for colony formation measures anchorage-independent *in vitro* transformation. The oncogene Bcl6 can confer anchorage-independent growth to immortalized cells [54]. To investigate the effects of EBNA3C on Bcl6-related transforming activity, the stable Bcl6 knock-down BJAB and LCL1 cells were generated with lentivirus transduction and puromycin selection (Fig 7A–7C). Soft agar assays were performed using the Bcl6 knock-down BJAB and LCL1 cells. The down-regulation of Bcl6 inhibited the ability of colony formation in BJAB cells (Fig 7D), but oppositely, the ability was enhanced in EBV-transformed LCLs (Fig 7E). The results indicate that EBV promotes transformation and anchorage-independent growth through the inhibition of Bcl6 expression.

### Down-regulation of Bcl6 facilitates G1-S transition through induction of CCND1 expression in EBV-transformed cells

**Fig 8:**
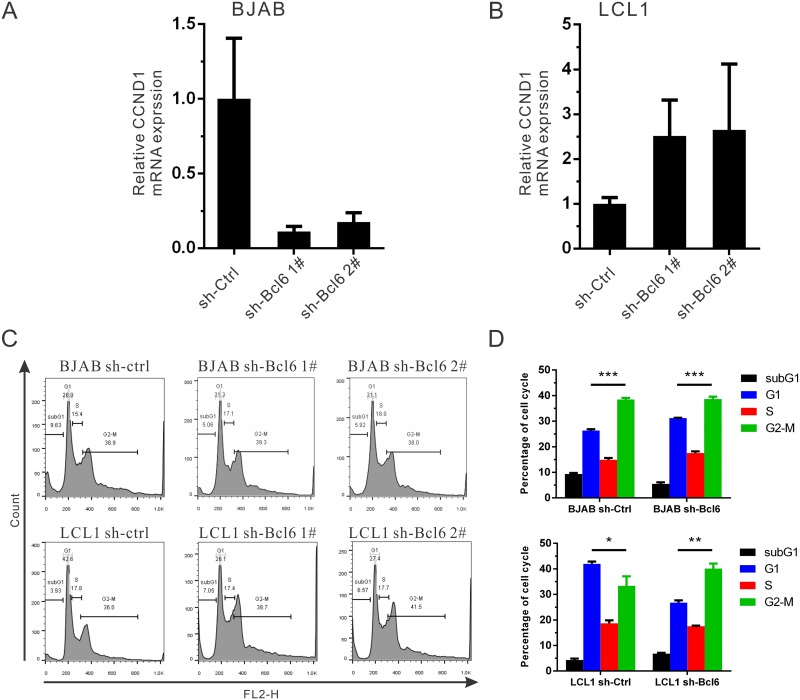
Down-regulation of Bcl6 facilitates G1-S transition through increasing CCND1 expression in EBV-transformed cells. A-B) The stable Bcl6 knock-down A) BJAB or B) LCL1 cells were collected and CCND1 mRNA expression was determined with Real-time PCR as mentioned earlier. C-D) The indicated stable cell lines were treated with PI staining buffer and analyzed by flow cytometry. Bar diagrams show the average values of two independent experiments.

Our previous study showed that EBNA3C could only stabilize Cyclin D1 (CCND1) protein, but not promote its transcription activity [40]. This suggests that other cellular factors may regulate CCND1 expression. Interestingly, CCND1 is induced by Bcl6 in human B-cells [55]. To further investigate the function of Bcl6 in cell cycle, we analyzed CCND1 mRNA expression in stable B-cells. The results show that CCND1 expression is also suppressed when Bcl6 is knocked-down in EBV-negative BJAB cells. However, its expression is upregulated in stable Bcl6 knock-down EBV-transformed LCL1 cells (Fig 8A and 8B). These results suggest that Bcl6 plays a critical role in controlling CCND1 mRNA expression in a B-cell background. The following cell cycle experiments also demonstrated that the upregulation of CCND1 through Bcl6 inhibition facilitates G1-S transition in EBV-transformed LCL1 cells, but not in the EBV-negative BJAB cells (Fig 8C and 8D). Similar results were observed with other sh-Bcl6 clones.

## Discussion

**Fig 9:**
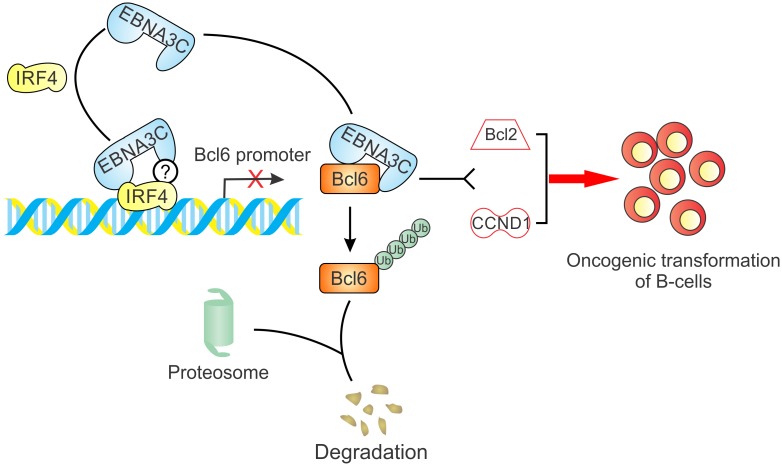
A schematic diagram shows a mechanism by which EBNA3C inhibits Bcl6 expression and further promotes the oncogenic cell growth and proliferation. EBNA3C interacts with Bcl6 protein and enhances its degradation through the ubiquitin-dependent pathway. Furthermore, EBNA3C also inhibits Bcl6 promoter transcription activity by recruiting IRF4. Finally, the EBNA3C-mediated Bcl6 downregulation contributes to cellular proliferation through regulation of its downstream signaling pathway (Bcl2 and CCND1), thus promoting oncogenic transformation in EBV infected cells.

Bcl6 is a nuclear phosphoprotein of the BTB/POZ/Zinc Finger (ZF) protein family, and functions as a transcription repressor to repress target genes by binding to specific DNA sequences and recruiting corepressors [8,56], including SMRT, MTA3, N-CoR and HDAC [57–60]. Bcl6 is indispensable for GC formation and somatic hypermutation (SHM) during B-cell development, thus chromosomal translocations and mutations of Bcl6 regulatory region lead to the deregulation of Bcl6 expression in about 40% DLBCL and 5–10% FL [46]. Although Bcl6 expression is associated with EBV latent antigen EBNA2 and LMP1, the reported conflicting results did not provide a reasonable explanation or a detailed mechanism on EBV-mediated Bcl6 degradation in B-cell lymphoma [37–39]. A recent study indicated that EBNA3C had no effects on Bcl6 expression, but a previous paper also showed that Bcl6 expression can be increased more than 10-fold in EBNA3C-deleted EBV infection [61,62]. Here, our data clearly show that Bcl6 expression can be down-regulated by EBNA3C specifically at transcriptional and post-transcriptional levels (Fig 9). This is different from the well-known Bcl6 translocations or mutations identified on the human genome associated with oncogenesis.

First of all, our results demonstrated that EBNA3C was specifically associated with Bcl6, and mediated Bcl6 protein degradation through the ubiquitin-proteasome dependent pathway. A previous study showed that Bcl6 protein can be targeted for degradation by cellular factor FBXO11 in DLBCL [63]. However, the role of EBNA3C on FBXO11-related Bcl6 stability is still unclear. It is possible that FBXO11 is the E3 ligase recruited by EBNA3C for Bcl6 degradation. In addition, the acetylation of Bcl6 within the PEST domain inactivates its function of recruiting co-repressors [54], and the activation of MAPK signaling pathway induces phosphorylation of Bcl6 followed by degradation through the ubiquitin-proteasome pathway [64]. Further studies are warranted to determine whether EBNA3C-mediated Bcl6 degradation is related to Bcl6 acetylation and phosphorylation.

BCL6 activity is also dysregulated by translocation or mutation in a remarkably high proportion of DLBCL and FL [65]. The chromosomal translocations of Bcl6 regulatory region referred to as promoter substitution, and frequent mutations of the 5’ noncoding region of Bcl6 result in its deregulated expression, suggesting a key role for Bcl6 in pathogenesis of B-cell lymphoma [12,13,66,67]. However, our results indicate that Bcl6 mRNA expression is down-regulated through EBNA3C-mediated inhibition of the transcription activity of Bcl6 promoter by recruiting another cellular factor IRF4. This is consistent with a previous study showing that the CD40 receptor signaling pathway leads to NF-κB-mediated IRF4 activation, and furthermore Bcl6 downregulation [47]. Meanwhile, we also showed that EBNA3C could interact with IRF4 and was critical for IRF4 stabilization [32]. This suggests that EBNA3C may mimic the activities of the CD40 ligand to induce NF-κB-IRF4 signaling pathway or enhance the stability of IRF4 protein directly to repress the transcription activity of the Bcl6 promoter. Moreover, EBNA3C was associated with IRF8 and mediated its destabilization and degradation [32]. Interestingly, IRF8 is the only transcriptional activator of Bcl6 to upregulate its mRNA expression in GC reaction [68]. Therefore, it is conceivable that EBNA3C may downregulate Bcl6 expression by activating CD40 signaling pathway as well as regulating IRF4/IRF8 stability.

The importance of Bcl6 function in GC B-cells is reflected in the multiple functional pathways it can regulate in the cell. To date, more than one thousand genes are found to be targeted by Bcl6 through binding on their promoters and further modulating the downstream signaling pathways during GC development, involved in cell apoptosis, cell cycle and cell differentiation [69,70]. Among the targeted proteins, Bcl2 is a critical anti-apoptosis protein and the direct target of Bcl6 that can interact with Miz1 and bind to Bcl2 promoter to inhibit Miz1-induced Bcl2 transcription activity in GC B-cells [52,53]. The dysregulation of Bcl6-mediated Bcl2 expression is often found in DLBCL and FL [2]. Our results show that Bcl2, a Bcl6 target protein, is regulated by EBNA3C and is increased in LCLs treated with a Bcl6 inhibitor. Thus EBNA3C can induce cell proliferation by degrading and inhibiting the expression of Bcl6 and so releasing the suppression of Bcl2, therefore activating the anti-apoptosis pathway for tumorigenesis. Moreover, CCND1, a direct target of Bcl6 in human B-cells, is de-repressed to promote G1-S transition in EBV-transformed LCLs. Whether other cyclin proteins are also under the control of Bcl6 is still unknown. Interestingly, several studies have shown that CCND2 is another target of Bcl6, but its expression is negatively correlated [71–75]. Activation-induced cytidine deaminase (AID) which is responsible for somatic hypermutation and class-switch recombination is also required in GC-derived lymphomas, and its expression is upregulated by EBNA3C in EBV-infected cells [61,76]. Bcl6 could promote AID expression by inhibiting miR-155 and mir-361, so how EBNA3C regulates AID expression without the help of Bcl6 needs to be further explored [2]. A recent study concluded that Bcl6 targeted genes in T follicular helper (Tfh) cells through analysis of its genome-wide occupancy and transcriptional regulatory networks [77]. The current development of Bcl6 small-molecular inhibitor indicates a huge potential for Bcl6 as a therapeutic target to treat human lymphomas [19]. However, the Bcl6-mediated regulatory networks are still unknown in EBV-transformed LCLs. Next, xenografts of LCLs in Bcl6 knock-out mice will further reveal the biological function of Bcl6 in EBV-related lymphomagenesis. However, a more efficient *in vivo* model will be necessary to uncover the crucial functions of EBNA3C or other latent antigens in GC reaction. In summary, the inhibition of Bcl6 expression by the essential EBV antigen EBNA3C may provide a novel insight into the current understanding of EBV contribution on lymphomagenesis by blocking GC reaction. Importantly, a number of EBV latent proteins are expressed in EBV infected cells, but how these latent proteins cooperate with each other to regulate B-cell development, or lead to B-cell lymphoma still needs further investigation. Nevertheless, our observations have implications for emerging strategies targeted at the EBV-associated cancers.

## Materials and methods

### Ethics statement

The University of Pennsylvania Immunology Core (HIC) provided us human peripheral blood mononuclear cells (PBMC) from different unidentified and healthy donors with written, informed consent. All the procedures were approved by the Institutional Review Board (IRB) and conducted according to the declarations of Helsinki protocols [36,78].

### Plasmids, cells and antibodies

Myc-tagged full-length EBNA3C or its truncations such as 1-365aa, 366-620aa, 621-992aa, and Flag-tagged IRF4 plasmids have been described previously [32]. Myc-tagged constructs expressing full length or DNA binding domain (DBD) mutant IRF4, and HA-tagged full length Bcl6, wild-type Bcl6 promoter plasmids [9,54] were kindly provided by Dr. Riccardo Dalla-Favera (Columbia University, New York, USA). HEK293 or HEK293T (human embryonic kidney cell line), Saos-2 (human osteosarcoma cell line), EBV-negative or -positive cells have been described earlier in detail [32,36]. MEF (mouse embryonic fibroblast cell line) was a gift from Xiaolu Yang (University of Pennsylvania) [79]. HEK293, HEK293T, Saos-2, MEF (p53-/-) and MEF (p53+/+) cells were grown in Dulbecco's modified Eagle's medium (DMEM), while B-cell lines were maintained in RPMI 1640 media. All the above-mentioned cells were incubated at 37°C in a humidified 5% CO_2_ environment.

The Bcl6 inhibitor (79–6) was purchased from EMD Millipore (Billerica, MA, USA). Bcl6 antibody (N-3) and Bcl2 antibody (C-2) were purchased from Santa Cruz biotechnology (Santa Cruz, CA, USA). Bcl6 antibody (ab19011) were purchased from Abcam (Cambridge, UK). Antibodies for IRF4, Ub, GAPDH have been described earlier [32]. Flag antibody (M2) was purchased from Sigma-Aldrich (St. Louis, MO, USA). Other antibodies to mouse anti-Myc (9E10), anti-HA (12CA5), anti-EBNA3C (A10) were prepared from hybridoma cultures and mentioned previously [80].

### Co-immunoprecipitation and western blot analyses

10 million transfected cells or 50 million B-cells were harvested, washed with ice-cold 1×PBS twice, lysed in 400μl ice-cold RIPA buffer [1% Nonidet P-40 (NP-40), 10 mM Tris (pH8.0), 2 mM EDTA, 150 mM NaCl, supplement with protease inhibitors (1 mM phenylmethylsulphonyl fluoride (PMSF), 1 μg/ml each aprotinin, pepstain and leupeptin]. Lysates were precleared with normal control serum plus 30 μl of a 2:1 mixture of Protein-A/G Sepharose beads (GE Healthcare Biosciences, Pittsburgh, PA) for 1 h at 4°C. Approximately 5% of the lysate was saved as input. About 1 μg of specific antibody was used to capture the protein of interest by overnight rotation at 4°C.

Input and IP samples were boiled in laemmli buffer, resolved on SDS-PAGE gel and transferred to a 0.45 μm nitrocellulose membrane. The membrane was blocked in 1×TBS-Tween with 5% w/v non-fat dry milk probed with appropriate primary antibody, subsequently incubated with corresponding secondary antibody, and visualized on a Licor Odyssey imager (LiCor Inc., Lincoln, NE). Image analysis and quantification measurements were performed using Image Quant application software (LiCor Inc., Lincoln, NE). The relative density (RD) of indicated proteins were shown.

### Immunofluorescence

HEK293T or Saos-2 cells plated on coverslips were transfected with expression plasmids or not as indicated. Forty eight hours post-transfection, cells were fixed by 4% paraformaldehyde (PFA) including 0.1% Triton X-100 for 15–20 mins at room temperature [81]. B-cells were air-dried and fixed similar to above. The fixed cells were washed with 1×PBS for three times, and 5% Bovine serum albumin (BSA) was used for blocking. EBNA3C and Bcl6 were detected by mouse anti-EBNA3C (A10) and rabbit anti-Bcl6 antibody, respectively. The slides were examined using an Olympus Fluoview 300 confocal microscope, and Images were analyzed by Fluoview software (Olympus Inc., Melville, NY).

### Dual-luciferase reporter assay

HEK293T cells were co-transfected with pLA/B9 plasmid (Bcl6 promoter, a gift from Dr. Riccardo Dalla-Favera) [47], pRL-TK (Promega, Madison, WI, USA), Myc-tagged EBNA3C, and control or Myc-tagged IRF4/IRF4-ΔDBD plasmids. Forty eight hours post-transfection, cells were harvested and the dual-luciferase reporter assay was performed according to the manufacture’s protocols (Promega, Madison, WI, USA). At the same time, the supernatant was collected and prepared for detection by western blot.

### RNA isolation and Real-time PCR

Cells were collected and washed with ice-cold 1×PBS prior to RNA isolation. Then total RNA extraction was performed using Trizol reagent (Invitrogen, Inc., Carlsbad, CA) and treated with Dnase I (Invitrogen, Inc., Carlsbad, CA), then cDNA was prepared with Superscript II reverse transcriptase kit (Invitrogen, Inc., Carlsbad, CA) according to the manufacturer’s protocol. Primers for GAPDH were 5′-TGCACCACCAACTGCTTAG-3′ and 5′-GATGCAGGGATGATGTTC-3′ [40]. Quantitative Real-time PCR analysis was performed by using SYBR green Real-time master mix (MJ Reserch Inc., Waltham, MA). The assays were performed in triplicate.

### Stability assay

Transfected HEK293T cells were treated with protein synthesis inhibitor cycloheximide (CalBiochem, Gibbstown, NJ) after 24 hours transfection as 40 μg/ml concentration. Cells were harvested after 16 hours incubation and lyse with RIPA buffer, then protein samples were quantitated and used for western blot analysis. Protein band intensities were quantified using Image Quant 3.0 software.

### Colony formation assay

10 million HEK293 or Saos-2 cells were transfected with control vector, Myc-EBNA3C, Myc-Bcl6 and GFP vector by electroporation and allowed to grow in DMEM supplemented with 1 mg/ml G418 (Sigma-Aldrich, St. Louis, MO, USA). After two weeks selection, GFP fluorescence of every plate was scanned by PhosphorImager (Molecular Dynamics, Piscataway, NJ) and the area of the colonies measured by using Image J software (Adobe Inc., San Jose, CA). Three independent experiments were performed.

### Lentiviral production and infection

The two sense strands of Bcl6 shRNA are 5’-tcgagtgctgttgacagtgagcgaGCCTGTTCTATAGCATCTTTAtagtgaagccacagatgtaTAAAGATGCTATAGAACAGGCgtgcctactgcctcggaa–3’ (sh-Bcl6-1), and 5’- tcgagtgctgttgacagtgagcgaCCACAGTGACAAACCCTACAAtagtgaagccacagatgtaTTGTAGGGTTTGTCACTGTGGgtgcctactgcctcggaa–3’ (sh-Bcl6-2), respectively. The upper-cases designate Bcl6 target sequences, while lower cases specify hairpin and enzyme sequences. These sense stranded oligos were annealed with their respective anti-sense stranded oligos, and then cloned into pGIPZ vector with Xho I and Mlu I restriction sites. Besides, a negative control was set using a sh-Ctrl plasmid including control shRNA sequence 5’-TCTCGCTTGGGCGAGAGTAAG–3’ (Dharmacon Research, Chicago, IL). Lentivirus production and transduction of B-cell lines has been described previously with a slight modification [32]. A pool of two shRNAs that targeted different regions of the Bcl6 mRNA were co-transfected to generated shRNA-expressing lentiviruses.

### Flow cytometry

The BJAB or LCL1 stable cell lines were generated according to the above-mentioned protocols. Approximately, 5 million BJAB or LCL1 stable cells were collected, fixed with 80% ethanol for 2 hours or overnight at -20°C, then washed with 1×PBS and incubated with PI staining buffer (0.5 mg/ml propidium iodide in 1×PBS, 50 μg/ml RNase A) for 30 minutes to 2 hours at room temperature. The indicated cells were washed with 1×PBS once, resuspended in 500 μl 1×PBS, and analyzed on FACS Calibur (Becton Dickinson, San Jose, CA, USA) using FlowJo software (TreeStar, San Carlos, CA, USA).

### Soft agar assays

The soft agar assays were performed using BJAB or LCL1 cells. Briefly, 1 ml of 0.5% agar in supplemented RPMI media was poured into 6-well plate and set aside to solidify. 0.5 ml 0.3% agar/medium containing 2×10^5^ cells was added to the previously plates as the middle layer. Then cells were covered with a top layer of another 1ml 0.5% agar/medium. After two weeks, colonies were stained with 0.005% crystal violet for 1 hour, and scanned using a Licor Odyssey system (LiCor Inc., Lincoln, NE). The number of colonies was counted using ImageJ software.

### Statistical analysis

Data represented here are the mean values with standard deviation (SD). The significance of differences in the mean values was calculated by performing 2-tailed student's t-test. P-value of <0.05 was considered as statistically significant in all our results (*P < 0.05; **P < 0.01; ***P < 0.001; NS, not significant).

### Accession numbers

Epstein-Barr virus (EBV) genome, strain B95-8-GenBank: V01555.2; EBNA3C (Human herpesvirus 4)-NCBI Reference Sequence: YP_401671.1; Bcl6 (Homo sapiens)-NCBI Reference Sequence: NM_001130845.1; IRF4 (Homo sapiens)-NCBI Reference Sequence: NM_002460.3; Bcl2 (Homo sapiens)-NCBI Reference Sequence: NM_000633.2; CCND1 (Homo sapiens)-NCBI Reference Sequence: NM_053056.2; p53 (Homo sapiens)-NCBI Reference Sequence: NM_000546.5.

## Supporting information

S1 Fig EBNA3C-mediated inhibition of Bcl6 promoter activity is independent of p53. MEF (p53-/-) and MEF (p53+/+) cells were transfected with wild-type Bcl6 promoter reporter plasmids in the presence of control vector or EBNA3C. At 48 hours post-transfection, luciferase activity was determined.
(TIF)

S2 Fig EBNA3C enhances Bcl2 expression through inhibition of Bcl6. A) LCL1 stable cells (sh-Ctrl, sh-Bcl6 1# and sh-E3C) were harvested and extracted total RNA. The levels of Bcl2 mRNA expression was detected with Real-Time PCR. B) Total RNAs from LCL1 stable cells (sh-Ctrl and sh-E3C) were isolated according to the manufacturer’s instructions and the levels of EBNA3C, Bcl6 and Bcl2 mRNA expression were quantified using Real-Time PCR.
(TIF)
