# Supplementary figures and images for: An essential EBV latent antigen 3C binds Bcl6 for targeted degradation and cell proliferation
Source: PLoS Pathog. 2017 Jul 24;13(7):e1006500. doi: 10.1371/journal.ppat.1006500 (PMC5524291; doi:10.1371/journal.ppat.1006500)

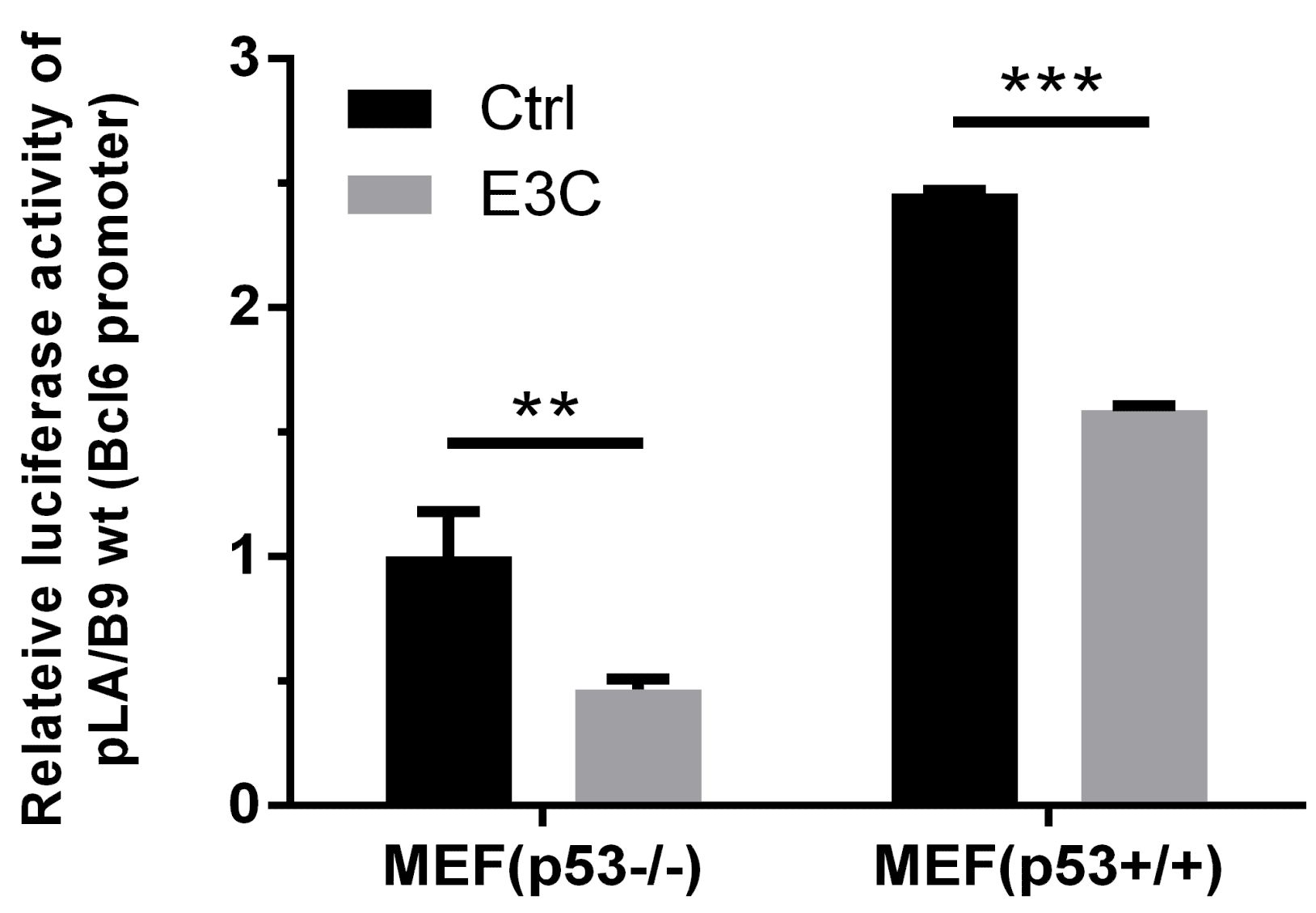

Supplement: S1 Fig — MEF (p53-/-) and MEF (p53+/+) cells were transfected with wild-type Bcl6 promoter reporter plasmids in the presence of control vector or EBNA3C. At 48 hours post-transfection, luciferase activity was determined. (TIF) [file ppat.1006500.s001.tif]

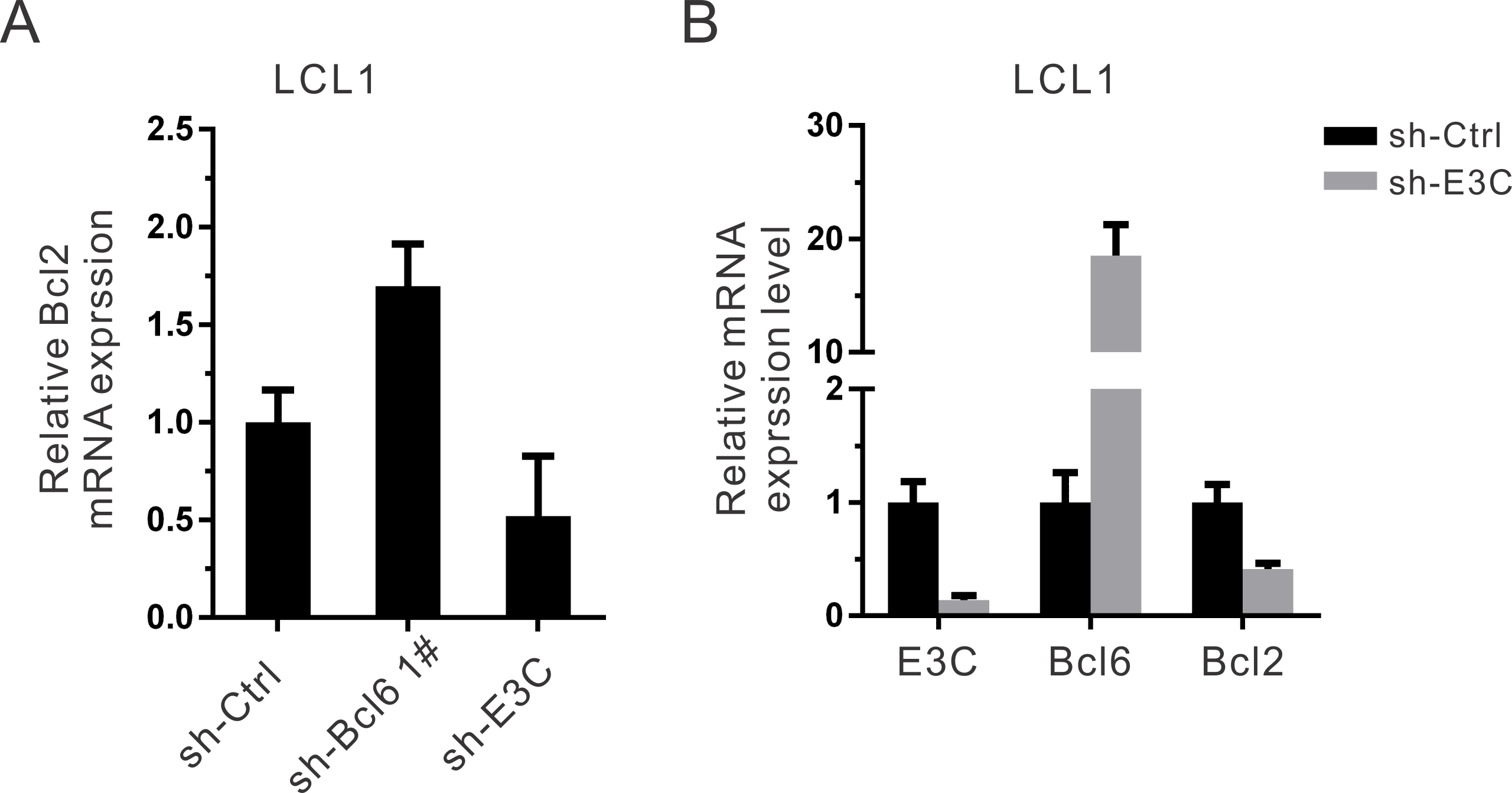

Supplement: S2 Fig — A) LCL1 stable cells (sh-Ctrl, sh-Bcl6 1# and sh-E3C) were harvested and extracted total RNA. The levels of Bcl2 mRNA expression was detected with Real-Time PCR. B) Total RNAs from LCL1 stable cells (sh-Ctrl and sh-E3C) were isolated according to the manufacturer’s instructions and the levels of EBNA3C, Bcl6 and Bcl2 mRNA expression were quantified using Real-Time PCR. (TIF) [file ppat.1006500.s002.tif]
